# Bis[tris­(ethane-1,2-diamine)nickel(II)] octa­cyanidomolybdate(IV) dihydrate

**DOI:** 10.1107/S1600536808024963

**Published:** 2008-08-13

**Authors:** Wen-Yan Liu, Hu Zhou, Ji-Xi Guo, Ai-Hua Yuan

**Affiliations:** a School of Materials Science and Engineering, Jiangsu University of Science and Technology, Zhenjiang 212003, People’s Republic of China; b Institute of Applied Chemistry, Xinjiang University, Urumqi 830046, Xinjiang, People’s Republic of China

## Abstract

The title complex, [Ni^II^(C_2_H_8_N_2_)_3_]_2_[Mo^IV^(CN)_8_]·2H_2_O, crystallized from a mixture of ethane-1,2-diamine (en), octa­cyano­molybdate(IV), [Mo(CN)_8_]^4−^, and the transition metal ion Ni^2+^. In the crystal structure, the Mo polyhedron has a square-anti­prismatic shape, while the geometry around the Ni atom is distorted octa­hedral. The complex ions and water mol­ecules are linked by hydrogen bonds.

## Related literature

For information on molybdenum–octa­cyanido complexes see: Mathonière *et al.* (2005 ▶); Przychodzeń *et al.* (2004 ▶); Zhou *et al.* (2008 ▶). For related literature, see: Chang *et al.* (2002 ▶); Leipoldt *et al.* (1974 ▶); Li *et al.* (2003 ▶); Podgajny *et al.* (2001 ▶); Przychodzeń *et al.* (2006 ▶); Sieklucka *et al.* (2002 ▶, 2005 ▶); Withers *et al.* (2005 ▶); Yuan *et al.* (2000 ▶).
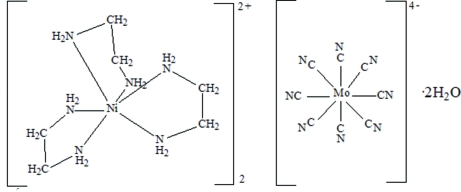


## Experimental

### 

#### Crystal data

[Ni(C_2_H_8_N_2_)_3_]_2_[Mo(CN)_8_]·2H_2_O
*M* *_r_* = 818.18
Monoclinic, 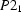

*a* = 10.1765 (3) Å
*b* = 12.2178 (2) Å
*c* = 15.8932 (3) Å
β = 106.683 (1)°
*V* = 1892.89 (7) Å^3^
*Z* = 2
Mo *K*α radiation
μ = 1.36 mm^−1^
*T* = 153 (2) K
0.30 × 0.26 × 0.24 mm

#### Data collection

Rigaku R-AXIS Spider diffractometer
Absorption correction: multi-scan (*ABSCOR*; Higashi, 1995 ▶) *T*_min_ = 0.67, *T*_max_ = 0.72
15991 measured reflections
6779 independent reflections
6292 reflections with *I* > 2σ(*I*)
*R*_int_ = 0.048

#### Refinement

*R*[*F* ^2^ > 2σ(*F* ^2^)] = 0.052
*wR*(*F* ^2^) = 0.122
*S* = 1.09
6779 reflections
433 parameters
1 restraint
H-atom parameters constrained
Δρ_max_ = 0.47 e Å^−3^
Δρ_min_ = −0.92 e Å^−3^
Absolute structure: Flack (1983 ▶), 2876 Friedel pairs
Flack parameter: 0.024 (19)

### 

Data collection: *RAPID-AUTO* (Rigaku, 2004 ▶); cell refinement: *RAPID-AUTO*; data reduction: *RAPID-AUTO*; program(s) used to solve structure: *SHELXTL* (Sheldrick, 2008 ▶); program(s) used to refine structure: *SHELXTL*; molecular graphics: *SHELXTL*; software used to prepare material for publication: *SHELXTL*.

## Supplementary Material

Crystal structure: contains datablocks global, I. DOI: 10.1107/S1600536808024963/br2077sup1.cif

Structure factors: contains datablocks I. DOI: 10.1107/S1600536808024963/br2077Isup2.hkl

Additional supplementary materials:  crystallographic information; 3D view; checkCIF report

## Figures and Tables

**Table 1:** Hydrogen-bond geometry (Å, °)

*D*—H⋯*A*	*D*—H	H⋯*A*	*D*⋯*A*	*D*—H⋯*A*
N9—H9*A*⋯N8^i^	0.92	2.27	3.140 (8)	159
N9—H9*B*⋯N3^ii^	0.92	2.41	3.179 (7)	141
N10—H10*A*⋯N3^iii^	0.92	2.20	3.111 (8)	170
N10—H10*B*⋯N5^iii^	0.92	2.58	3.252 (8)	130
N11—H11*A*⋯N3^iii^	0.92	2.37	3.219 (8)	153
N12—H12*A*⋯N7^iv^	0.92	2.35	3.141 (8)	144
N12—H12*B*⋯N5^iii^	0.92	2.25	3.126 (8)	159
N13—H13*A*⋯N7^iv^	0.92	2.53	3.434 (8)	167
N13—H13*B*⋯N3^ii^	0.92	2.25	3.056 (8)	146
N14—H14*A*⋯N8^i^	0.92	2.24	3.066 (8)	149
N14—H14*B*⋯O3^iii^	0.92	2.18	3.099 (15)	176
N15—H15*A*⋯O1	0.92	2.51	3.290 (16)	143
N15—H15*B*⋯O4^v^	0.92	2.48	3.342 (16)	156
N16—H16*A*⋯O4	0.92	2.18	3.023 (16)	152
N17—H17*A*⋯O4	0.92	2.17	3.040 (17)	157
N17—H17*B*⋯O5^v^	0.92	2.28	3.097 (14)	148
N18—H18*A*⋯N6^i^	0.92	2.26	3.177 (10)	178
N18—H18*B*⋯N4	0.92	2.40	3.216 (8)	148
N19—H19*A*⋯N2^i^	0.92	2.63	3.246 (8)	125
N19—H19*B*⋯O4^v^	0.92	2.30	3.187 (15)	163
N20—H20*A*⋯O3	0.92	2.25	3.165 (12)	172
N20—H20*B*⋯N4	0.92	2.43	3.306 (10)	159

Symmetry codes: (i) 
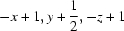
; (ii) 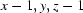; (iii) 
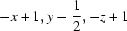
; (iv) 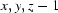; (v) 
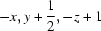
.

**Fig. 1.**
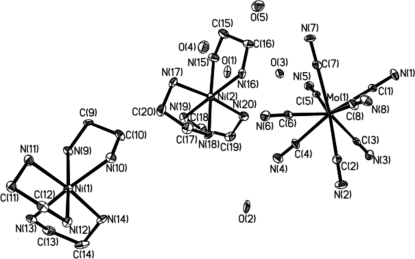
ORTEP view of the title complex showing 30% probability thermal motion ellipsoid. Hydrogen atoms have been omitted for clarity.

**Fig. 2.**
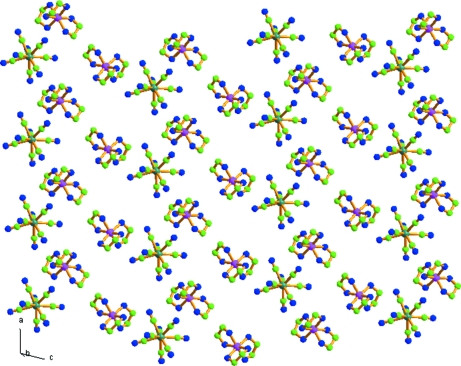
Packing diagram of the title complex. Water molecules are omitted for clarity.

## supplementary crystallographic information

### Comment

Recently, there is a continuing interest in donor-acceptor systems based on octacyano-complexes [*M*(CN)_8_]^3-/4-^ (*M* = Mo, W and Nb) as building blocks for constructing either ion-paired or cyano-bridged bimetallic assemblies because of their unique structures and physicochemical properties (Przychodzeń *et al.*, 2006; Sieklucka *et al.*, 2005; Sieklucka *et al.*, 2002). While it was very difficult to crystallize octacyanometalate networks by slow diffusion or hydrothermal methods, we began to explore the utility of amine ligands to direct and limit the number of cyano linkages formed between transition metal centers. Bidentate amines such as 1,2-diaminoethane (en), 1,2-diaminopropane (pn), and 1,3-propanediamine (tn), are found extensive use in the preparation of so-called 'expanded Prussian blue solids', but only several octacyanometalate derivatives are known up to date (Li *et al.*, 2003; Podgajny *et al.*, 2001; Withers *et al.*, 2005; Chang *et al.*, 2002; Yuan *et al.*, 2000).

Each Mo atom is eight-coordinated, where all eight cyanide groups are terminal ones (Fig. 1). The Mo(IV) polyhedron has a square-antiprismatic shape (D*_4_*_d_ symmetry). The octacyanomolybdate moiety [Mo(CN)_8_]^4-^ is characterized by the average Mo—C distance of 2.161 Å and practically linear Mo—CN bonds with the bond angles ranging from 176.8 (4)° to 179.6 (4)°. Crystal structure data of octacyanometalate moiety Mo(IV) (SAPR-8, D*_4_*_d _symmetry) is in very good agreement with the great majority of mononuclear [Mo(CN)_8_]^4-^ complexes (Zhou *et al.*, 2008; Mathonière *et al.*, 2005; Przychodzeń *et al.*, 2004).

In [Ni(en)_3_]^2+^cation, the geometry around Ni(1) is octahedron (D*_3_* symmetry). The average Ni(1)—N bond distance for the [Ni(1)(en)_3_]^2+^centers is 2.122 Å. The octahedral environment of [Ni(en)_3_]^2+^is comparable with those found for other Ni complexes previously reported with amine and polyamine ligands (Withers *et al.*, 2005; Chang *et al.*, 2002; Yuan *et al.*, 2000).

The striking aspect of the structure lies in the coordination environment of [Ni(en)_3_]^2+^cation. Each Ni atom chelates to three en molecules, and no positions are provided for CN groups of [Mo(CN)_8_]^4-^ thus avoiding the formation of cyano-bridge. Packing diagram of the title complex is shown in figure 2. There are additional water molecules (O1, O2, O3, O4, O5) in the structure, which might be connected *via* difference map.

### Experimental

For the preparation of the title complex, pale yellow crystals of suitable for X-ray single-crystal structure determination were grown at room temperature by slow diffusion of an aqueous solution of NiCl_2_.H_2_O (0.2 mmol) and ethane-1,2-amine (en, 0.6 mmol) and anaqueous solution of K_4_[Mo(CN)_8_].2H_2_O (Leipoldt *et al.*, 1974) (0.1 mmol) for six weeks. The resulting crystals were collected, washed with H_2_O and dried in air. *Anal*. Calc. for C_20_H_52_MoN_20_Ni_2_O_2_: C, 29.36; H, 6.41; N, 34.24; Ni, 14.35; Mo, 11.73. Found: C, 29.50; H, 6.38; N, 34.11; Ni, 14.40; Mo, 11.77.

### Refinement

All non-H atoms were refined anisotropically. The H atoms on nitrogen atoms were located from the difference Fourier maps, and the H atoms of water molecules were not located in the difference map and placed in theory positions.

### Crystal data

**Table:**

[Ni(C_2_H_8_N_2_)_3_]_2_[Mo(CN)_8_]·2H_2_O	*F*_000_ = 852
*M**_r_* = 818.18	*D*_x_ = 1.435 Mg m^−^^3^
Monoclinic, *P*2_1_	Mo *K*α radiation λ = 0.71073 Å
Hall symbol: P 2yb	Cell parameters from 4517 reflections
*a* = 10.1765 (3) Å	θ = 2.1–25.5º
*b* = 12.2178 (2) Å	µ = 1.36 mm^−^^1^
*c* = 15.8932 (3) Å	*T* = 153 (2) K
β = 106.6830 (10)º	Pale, yellow
*V* = 1892.89 (7) Å^3^	0.30 × 0.26 × 0.24 mm
*Z* = 2	

### Data collection

**Table:**

Bruker SMART APEX CCD diffractometer	6779 independent reflections
Radiation source: sealed tube	6292 reflections with *I* > \2s(*I*)
Monochromator: graphite	*R*_int_ = 0.048
*T* = 153(2) K	θ_max_ = 26.0º
φ and ω scans	θ_min_ = 3.2º
Absorption correction: multi-scan(SADABS; Bruker, 2000)	*h* = −12→12
*T*_min_ = 0.67, *T*_max_ = 0.72	*k* = −15→13
15991 measured reflections	*l* = −19→19

### Refinement

**Table:**

Refinement on *F*^2^	Hydrogen site location: inferred from neighbouring sites
Least-squares matrix: full	H-atom parameters constrained
*R*[*F*^2^ > 2σ(*F*^2^)] = 0.052	*w* = 1/[σ^2^(*F*_o_^2^) + (0.07*P*)^2^ + 1.99*P*] where *P* = (*F*_o_^2^ + 2*F*_c_^2^)/3
*wR*(*F*^2^) = 0.122	(Δ/σ)_max_ < 0.001
*S* = 1.09	Δρ_max_ = 0.48 e Å^−^^3^
6779 reflections	Δρ_min_ = −0.92 e Å^−^^3^
433 parameters	Extinction correction: none
1 restraint	Absolute structure: Flack (1983), 2876 Friedel pairs
Primary atom site location: structure-invariant direct methods	Flack parameter: 0.024 (19)
Secondary atom site location: difference Fourier map	

### Special details

**Table:**

Geometry. All e.s.d.'s (except the e.s.d. in the dihedral angle between two l.s. planes) are estimated using the full covariance matrix. The cell e.s.d.'s are taken into account individually in the estimation of e.s.d.'s in distances, angles and torsion angles; correlations between e.s.d.'s in cell parameters are only used when they are defined by crystal symmetry. An approximate (isotropic) treatment of cell e.s.d.'s is used for estimating e.s.d.'s involving l.s. planes.
Refinement. Refinement of *F*^2^ against ALL reflections. The weighted *R*-factor *wR* and goodness of fit *S* are based on *F*^2^, conventional *R*-factors *R* are based on *F*, with *F* set to zero for negative *F*^2^. The threshold expression of *F*^2^ > σ(*F*^2^) is used only for calculating *R*-factors(gt) *etc*. and is not relevant to the choice of reflections for refinement. *R*-factors based on *F*^2^ are statistically about twice as large as those based on *F*, and *R*- factors based on ALL data will be even larger.

### Fractional atomic coordinates and isotropic or equivalent isotropic displacement parameters (Å^2^)

**Table:**

	*x*	*y*	*z*	*U*_iso_*/*U*_eq_	Occ. (<1)
C1	0.7021 (6)	0.2552 (5)	0.9316 (4)	0.0195 (12)	
C2	0.8354 (6)	0.2524 (6)	0.7410 (4)	0.0241 (13)	
C3	0.8353 (6)	0.3887 (5)	0.8634 (4)	0.0189 (12)	
C4	0.6661 (7)	0.4211 (6)	0.7016 (4)	0.0278 (14)	
C5	0.5555 (6)	0.4260 (6)	0.8268 (4)	0.0199 (12)	
C6	0.5563 (6)	0.2339 (6)	0.6619 (4)	0.0289 (14)	
C7	0.4715 (7)	0.2262 (5)	0.7999 (4)	0.0264 (14)	
C8	0.7056 (7)	0.1166 (6)	0.8070 (4)	0.0287 (14)	
C9	0.0508 (5)	0.2749 (6)	0.1559 (4)	0.0248 (14)	
H9C	−0.0161	0.2162	0.1310	0.030*	
H9D	0.0106	0.3235	0.1919	0.030*	
C10	0.1825 (7)	0.2257 (6)	0.2122 (4)	0.0271 (14)	
H10C	0.2469	0.2845	0.2406	0.032*	
H10D	0.1638	0.1798	0.2588	0.032*	
C11	0.1076 (6)	0.0854 (5)	−0.1054 (4)	0.0243 (13)	
H11C	0.1146	0.1382	−0.1511	0.029*	
H11D	0.0442	0.0259	−0.1338	0.029*	
C12	0.2488 (7)	0.0386 (5)	−0.0584 (4)	0.0253 (14)	
H12C	0.2398	−0.0189	−0.0165	0.030*	
H12D	0.2896	0.0050	−0.1017	0.030*	
C13	0.2762 (8)	0.4470 (6)	−0.0335 (5)	0.0325 (15)	
H13C	0.2183	0.4868	−0.0032	0.039*	
H13D	0.2901	0.4949	−0.0805	0.039*	
C14	0.4120 (7)	0.4211 (6)	0.0306 (6)	0.0356 (17)	
H14C	0.4735	0.3876	−0.0005	0.043*	
H14D	0.4555	0.4891	0.0594	0.043*	
C15	0.0575 (8)	0.5073 (7)	0.6690 (5)	0.0371 (17)	
H15C	−0.0142	0.4691	0.6229	0.045*	
H15D	0.0181	0.5276	0.7170	0.045*	
C16	0.1786 (7)	0.4333 (7)	0.7037 (5)	0.0345 (16)	
H16C	0.2331	0.4602	0.7620	0.041*	
H16D	0.1453	0.3590	0.7117	0.041*	
C17	0.3069 (7)	0.4275 (7)	0.4299 (5)	0.0364 (17)	
H17C	0.3547	0.4193	0.3842	0.044*	
H17D	0.3046	0.3551	0.4575	0.044*	
C18	0.3033 (7)	0.8036 (6)	0.5511 (5)	0.0324 (15)	
H18C	0.2475	0.8442	0.5825	0.039*	
H18D	0.3332	0.8563	0.5129	0.039*	
C19	0.4256 (7)	0.7580 (6)	0.6159 (5)	0.0330 (17)	
H19C	0.4603	0.8126	0.6631	0.040*	
H19D	0.4981	0.7460	0.5867	0.040*	
C20	0.1658 (7)	0.4669 (7)	0.3898 (5)	0.0370 (17)	
H20C	0.1142	0.4126	0.3466	0.044*	
H20D	0.1680	0.5366	0.3586	0.044*	
Mo1	0.66541 (4)	0.28850 (4)	0.79163 (3)	0.01679 (12)	
N1	0.7199 (6)	0.2377 (5)	1.0040 (4)	0.0301 (12)	
N2	0.9261 (6)	0.2312 (6)	0.7138 (4)	0.0340 (13)	
N3	0.9209 (5)	0.4433 (5)	0.9004 (4)	0.0268 (12)	
N4	0.6622 (7)	0.4940 (6)	0.6546 (4)	0.0396 (15)	
N5	0.4988 (6)	0.5007 (5)	0.8419 (4)	0.0284 (12)	
N6	0.5011 (6)	0.2020 (7)	0.5923 (4)	0.0424 (17)	
N7	0.3659 (6)	0.1947 (6)	0.8043 (4)	0.0324 (14)	
N8	0.7278 (6)	0.0243 (5)	0.8114 (4)	0.0334 (13)	
N9	0.0806 (5)	0.3382 (4)	0.0843 (3)	0.0198 (10)	
H9A	0.1197	0.4044	0.1051	0.024*	
H9B	0.0011	0.3511	0.0401	0.024*	
N10	0.2435 (5)	0.1588 (5)	0.1566 (3)	0.0233 (11)	
H10A	0.2006	0.0918	0.1463	0.028*	
H10B	0.3351	0.1474	0.1844	0.028*	
N11	0.0556 (6)	0.1416 (5)	−0.0374 (3)	0.0250 (12)	
H11A	0.0319	0.0911	−0.0014	0.030*	
H11B	−0.0201	0.1836	−0.0637	0.030*	
N12	0.3389 (5)	0.1272 (4)	−0.0107 (3)	0.0205 (11)	
H12A	0.3809	0.1614	−0.0476	0.025*	
H12B	0.4059	0.0983	0.0358	0.025*	
N13	0.2079 (6)	0.3480 (4)	−0.0718 (4)	0.0240 (11)	
H13A	0.2509	0.3175	−0.1097	0.029*	
H13B	0.1179	0.3619	−0.1020	0.029*	
N14	0.3906 (5)	0.3450 (5)	0.0966 (4)	0.0260 (11)	
H14A	0.3734	0.3833	0.1421	0.031*	
H14B	0.4679	0.3031	0.1189	0.031*	
N15	0.1003 (6)	0.6013 (5)	0.6341 (4)	0.0349 (14)	
H15A	0.1393	0.6491	0.6791	0.042*	
H15B	0.0250	0.6352	0.5971	0.042*	
N16	0.2683 (6)	0.4264 (5)	0.6456 (4)	0.0305 (13)	
H16A	0.2469	0.3647	0.6111	0.037*	
H16B	0.3583	0.4210	0.6789	0.037*	
N17	0.0968 (5)	0.4836 (5)	0.4593 (4)	0.0298 (13)	
H17A	0.0728	0.4175	0.4784	0.036*	
H17B	0.0188	0.5252	0.4384	0.036*	
N18	0.3814 (5)	0.5073 (5)	0.4973 (4)	0.0286 (13)	
H18A	0.4137	0.5642	0.4709	0.034*	
H18B	0.4550	0.4738	0.5363	0.034*	
N19	0.2186 (6)	0.7195 (6)	0.4965 (4)	0.0381 (15)	
H19A	0.2433	0.7116	0.4455	0.046*	
H19B	0.1280	0.7405	0.4817	0.046*	
N20	0.4008 (6)	0.6538 (6)	0.6563 (4)	0.0379 (15)	
H20A	0.3753	0.6677	0.7063	0.046*	
H20B	0.4798	0.6126	0.6716	0.046*	
Ni1	0.22011 (7)	0.24205 (6)	0.03593 (5)	0.01682 (16)	
Ni2	0.24281 (8)	0.56721 (7)	0.56448 (5)	0.02290 (19)	
O1	0.1546 (14)	0.8476 (13)	0.7207 (9)	0.043 (5)	0.395 (18)
H1B	0.2345	0.8488	0.7568	0.052*	0.395 (18)
H1C	0.1500	0.8944	0.6804	0.052*	0.395 (18)
O2	0.7361 (13)	0.6346 (12)	0.5413 (9)	0.041 (5)	0.410 (18)
H2D	0.6725	0.6587	0.5612	0.050*	0.410 (18)
H2A	0.7418	0.6743	0.4986	0.050*	0.410 (18)
O3	0.3445 (12)	0.7066 (11)	0.8378 (7)	0.032 (4)	0.392 (16)
H3B	0.2721	0.7450	0.8289	0.038*	0.392 (16)
H3C	0.3339	0.6467	0.8623	0.038*	0.392 (16)
O4	0.1011 (13)	0.2511 (13)	0.5281 (9)	0.044 (5)	0.399 (17)
H4A	0.1079	0.1929	0.5583	0.052*	0.399 (17)
H4C	0.1316	0.2397	0.4845	0.052*	0.399 (17)
O5	0.1340 (13)	0.1064 (12)	0.6782 (9)	0.042 (4)	0.404 (17)
H5A	0.0736	0.1221	0.7037	0.050*	0.404 (17)
H5C	0.2134	0.1161	0.7136	0.050*	0.404 (17)

### Atomic displacement parameters (Å^2^)

**Table:**

	*U*^11^	*U*^22^	*U*^33^	*U*^12^	*U*^13^	*U*^23^
C1	0.023 (3)	0.018 (3)	0.018 (3)	−0.005 (2)	0.007 (2)	0.000 (2)
C2	0.022 (3)	0.021 (3)	0.027 (3)	−0.008 (2)	0.004 (2)	0.001 (2)
C3	0.027 (3)	0.010 (3)	0.020 (3)	−0.002 (2)	0.007 (2)	0.007 (2)
C4	0.034 (3)	0.026 (4)	0.021 (3)	−0.006 (3)	0.003 (3)	0.010 (3)
C5	0.015 (2)	0.024 (3)	0.022 (3)	−0.002 (2)	0.007 (2)	0.005 (2)
C6	0.026 (3)	0.036 (4)	0.024 (3)	−0.014 (3)	0.004 (3)	0.008 (3)
C7	0.031 (3)	0.019 (3)	0.028 (3)	−0.008 (3)	0.006 (3)	0.004 (3)
C8	0.038 (4)	0.026 (4)	0.024 (3)	0.007 (3)	0.013 (3)	0.006 (3)
C9	0.013 (2)	0.037 (4)	0.029 (3)	0.001 (2)	0.013 (2)	0.009 (3)
C10	0.040 (4)	0.024 (4)	0.020 (3)	0.007 (3)	0.012 (3)	0.007 (3)
C11	0.029 (3)	0.023 (3)	0.021 (3)	−0.002 (2)	0.007 (2)	−0.007 (2)
C12	0.036 (3)	0.017 (3)	0.026 (3)	0.002 (2)	0.014 (3)	0.002 (2)
C13	0.039 (4)	0.024 (4)	0.041 (4)	−0.006 (3)	0.021 (3)	0.002 (3)
C14	0.025 (3)	0.027 (4)	0.062 (5)	−0.006 (3)	0.023 (3)	0.000 (3)
C15	0.042 (4)	0.038 (4)	0.037 (4)	0.008 (3)	0.020 (3)	0.013 (3)
C16	0.032 (4)	0.036 (4)	0.038 (4)	−0.008 (3)	0.014 (3)	0.009 (3)
C17	0.034 (4)	0.042 (4)	0.035 (4)	0.015 (3)	0.014 (3)	−0.011 (3)
C18	0.043 (4)	0.021 (4)	0.034 (3)	0.001 (3)	0.013 (3)	−0.010 (3)
C19	0.032 (3)	0.030 (4)	0.038 (4)	−0.016 (3)	0.010 (3)	−0.022 (3)
C20	0.031 (3)	0.045 (5)	0.031 (4)	0.003 (3)	0.001 (3)	−0.009 (3)
Mo1	0.0170 (2)	0.0178 (2)	0.0146 (2)	−0.0064 (2)	0.00296 (16)	0.0014 (2)
N1	0.044 (3)	0.021 (3)	0.023 (3)	−0.003 (3)	0.006 (2)	0.007 (2)
N2	0.027 (3)	0.042 (4)	0.038 (3)	−0.014 (3)	0.016 (2)	−0.009 (3)
N3	0.026 (3)	0.023 (3)	0.029 (3)	0.003 (2)	0.004 (2)	−0.001 (2)
N4	0.039 (3)	0.049 (4)	0.030 (3)	−0.013 (3)	0.009 (3)	0.007 (3)
N5	0.028 (3)	0.030 (3)	0.028 (3)	−0.005 (2)	0.009 (2)	−0.003 (2)
N6	0.028 (3)	0.073 (5)	0.024 (3)	−0.015 (3)	0.003 (2)	−0.003 (3)
N7	0.025 (3)	0.047 (4)	0.028 (3)	−0.020 (3)	0.011 (2)	−0.007 (3)
N8	0.045 (3)	0.023 (3)	0.036 (3)	−0.005 (3)	0.018 (3)	0.005 (2)
N9	0.028 (3)	0.014 (2)	0.020 (3)	0.003 (2)	0.011 (2)	0.000 (2)
N10	0.027 (3)	0.020 (3)	0.024 (3)	0.001 (2)	0.009 (2)	−0.003 (2)
N11	0.028 (3)	0.026 (3)	0.020 (3)	−0.001 (2)	0.007 (2)	−0.006 (2)
N12	0.020 (2)	0.017 (3)	0.024 (3)	0.003 (2)	0.005 (2)	−0.003 (2)
N13	0.032 (3)	0.019 (3)	0.026 (3)	0.002 (2)	0.017 (2)	0.004 (2)
N14	0.014 (2)	0.028 (3)	0.036 (3)	−0.003 (2)	0.007 (2)	−0.005 (2)
N15	0.041 (3)	0.036 (4)	0.032 (3)	−0.014 (3)	0.018 (3)	−0.007 (3)
N16	0.025 (3)	0.042 (4)	0.021 (3)	0.000 (2)	−0.001 (2)	0.000 (2)
N17	0.017 (2)	0.037 (3)	0.030 (3)	0.000 (2)	−0.001 (2)	−0.005 (2)
N18	0.010 (2)	0.048 (4)	0.028 (3)	0.002 (2)	0.007 (2)	−0.007 (3)
N19	0.038 (3)	0.047 (4)	0.028 (3)	−0.001 (3)	0.006 (3)	0.006 (3)
N20	0.035 (3)	0.060 (5)	0.020 (3)	−0.011 (3)	0.011 (3)	−0.014 (3)
Ni1	0.0161 (3)	0.0141 (3)	0.0214 (4)	0.0003 (3)	0.0072 (3)	−0.0002 (3)
Ni2	0.0217 (4)	0.0281 (4)	0.0188 (4)	−0.0018 (3)	0.0057 (3)	−0.0039 (3)
O1	0.038 (7)	0.048 (9)	0.032 (7)	0.007 (6)	−0.008 (6)	0.005 (6)
O2	0.032 (6)	0.044 (9)	0.034 (7)	0.002 (5)	−0.014 (5)	0.015 (6)
O3	0.029 (6)	0.046 (8)	0.024 (6)	0.017 (5)	0.014 (5)	0.006 (5)
O4	0.038 (7)	0.044 (9)	0.044 (8)	−0.001 (6)	0.003 (6)	0.005 (6)
O5	0.037 (7)	0.051 (9)	0.038 (7)	−0.012 (6)	0.012 (6)	−0.013 (6)

### Geometric parameters (Å, °)

**Table:**

C1—N1	1.133 (8)	C19—N20	1.479 (10)
C1—Mo1	2.185 (6)	C19—H19C	0.9900
C2—N2	1.155 (9)	C19—H19D	0.9900
C2—Mo1	2.153 (6)	C20—N17	1.483 (9)
C3—N3	1.121 (8)	C20—H20C	0.9900
C3—Mo1	2.158 (6)	C20—H20D	0.9900
C4—N4	1.156 (9)	N9—Ni1	2.148 (5)
C4—Mo1	2.162 (6)	N9—H9A	0.9200
C5—N5	1.142 (9)	N9—H9B	0.9200
C5—Mo1	2.178 (7)	N10—Ni1	2.123 (6)
C6—N6	1.156 (9)	N10—H10A	0.9200
C6—Mo1	2.148 (7)	N10—H10B	0.9200
C7—N7	1.162 (9)	N11—Ni1	2.132 (5)
C7—Mo1	2.154 (6)	N11—H11A	0.9200
C8—N8	1.148 (9)	N11—H11B	0.9200
C8—Mo1	2.141 (7)	N12—Ni1	2.120 (5)
C9—N9	1.478 (8)	N12—H12A	0.9200
C9—C10	1.506 (8)	N12—H12B	0.9200
C9—H9C	0.9900	N13—Ni1	2.121 (5)
C9—H9D	0.9900	N13—H13A	0.9200
C10—N10	1.466 (8)	N13—H13B	0.9200
C10—H10C	0.9900	N14—Ni1	2.135 (5)
C10—H10D	0.9900	N14—H14A	0.9200
C11—N11	1.498 (8)	N14—H14B	0.9200
C11—C12	1.528 (9)	N15—Ni2	2.103 (6)
C11—H11C	0.9900	N15—H15A	0.9200
C11—H11D	0.9900	N15—H15B	0.9200
C12—N12	1.479 (8)	N16—Ni2	2.120 (6)
C12—H12C	0.9900	N16—H16A	0.9200
C12—H12D	0.9900	N16—H16B	0.9200
C13—N13	1.440 (9)	N17—Ni2	2.149 (5)
C13—C14	1.496 (11)	N17—H17A	0.9200
C13—H13C	0.9900	N17—H17B	0.9200
C13—H13D	0.9900	N18—Ni2	2.129 (5)
C14—N14	1.465 (9)	N18—H18A	0.9200
C14—H14C	0.9900	N18—H18B	0.9200
C14—H14D	0.9900	N19—Ni2	2.131 (7)
C15—N15	1.399 (9)	N19—H19A	0.9200
C15—C16	1.500 (10)	N19—H19B	0.9200
C15—H15C	0.9900	N20—Ni2	2.118 (6)
C15—H15D	0.9900	N20—H20A	0.9200
C16—N16	1.476 (9)	N20—H20B	0.9200
C16—H16C	0.9900	O1—H1B	0.8500
C16—H16D	0.9900	O1—H1C	0.8501
C17—C20	1.474 (10)	O2—H2D	0.8499
C17—N18	1.485 (9)	O2—H2A	0.8500
C17—H17C	0.9900	O3—H3B	0.8500
C17—H17D	0.9900	O3—H3C	0.8500
C18—N19	1.457 (9)	O4—H4A	0.8499
C18—C19	1.479 (10)	O4—H4C	0.8500
C18—H18C	0.9900	O5—H5A	0.8500
C18—H18D	0.9900	O5—H5C	0.8500
			
N1—C1—Mo1	179.4 (5)	C9—N9—Ni1	107.0 (4)
N2—C2—Mo1	178.8 (6)	C9—N9—H9A	110.3
N3—C3—Mo1	177.9 (5)	Ni1—N9—H9A	110.3
N4—C4—Mo1	177.1 (7)	C9—N9—H9B	110.3
N5—C5—Mo1	176.7 (6)	Ni1—N9—H9B	110.3
N6—C6—Mo1	177.7 (7)	H9A—N9—H9B	108.6
N7—C7—Mo1	178.6 (6)	C10—N10—Ni1	108.7 (4)
N8—C8—Mo1	176.8 (6)	C10—N10—H10A	110.0
N9—C9—C10	108.7 (5)	Ni1—N10—H10A	110.0
N9—C9—H9C	109.9	C10—N10—H10B	110.0
C10—C9—H9C	109.9	Ni1—N10—H10B	110.0
N9—C9—H9D	109.9	H10A—N10—H10B	108.3
C10—C9—H9D	109.9	C11—N11—Ni1	105.9 (4)
H9C—C9—H9D	108.3	C11—N11—H11A	110.6
N10—C10—C9	108.9 (5)	Ni1—N11—H11A	110.6
N10—C10—H10C	109.9	C11—N11—H11B	110.6
C9—C10—H10C	109.9	Ni1—N11—H11B	110.6
N10—C10—H10D	109.9	H11A—N11—H11B	108.7
C9—C10—H10D	109.9	C12—N12—Ni1	109.4 (4)
H10C—C10—H10D	108.3	C12—N12—H12A	109.8
N11—C11—C12	107.2 (5)	Ni1—N12—H12A	109.8
N11—C11—H11C	110.3	C12—N12—H12B	109.8
C12—C11—H11C	110.3	Ni1—N12—H12B	109.8
N11—C11—H11D	110.3	H12A—N12—H12B	108.2
C12—C11—H11D	110.3	C13—N13—Ni1	105.2 (4)
H11C—C11—H11D	108.5	C13—N13—H13A	110.7
N12—C12—C11	109.5 (5)	Ni1—N13—H13A	110.7
N12—C12—H12C	109.8	C13—N13—H13B	110.7
C11—C12—H12C	109.8	Ni1—N13—H13B	110.7
N12—C12—H12D	109.8	H13A—N13—H13B	108.8
C11—C12—H12D	109.8	C14—N14—Ni1	108.6 (4)
H12C—C12—H12D	108.2	C14—N14—H14A	110.0
N13—C13—C14	110.4 (6)	Ni1—N14—H14A	110.0
N13—C13—H13C	109.6	C14—N14—H14B	110.0
C14—C13—H13C	109.6	Ni1—N14—H14B	110.0
N13—C13—H13D	109.6	H14A—N14—H14B	108.3
C14—C13—H13D	109.6	C15—N15—Ni2	112.7 (5)
H13C—C13—H13D	108.1	C15—N15—H15A	109.0
N14—C14—C13	108.9 (5)	Ni2—N15—H15A	109.0
N14—C14—H14C	109.9	C15—N15—H15B	109.0
C13—C14—H14C	109.9	Ni2—N15—H15B	109.0
N14—C14—H14D	109.9	H15A—N15—H15B	107.8
C13—C14—H14D	109.9	C16—N16—Ni2	110.1 (5)
H14C—C14—H14D	108.3	C16—N16—H16A	109.6
N15—C15—C16	108.9 (6)	Ni2—N16—H16A	109.6
N15—C15—H15C	109.9	C16—N16—H16B	109.6
C16—C15—H15C	109.9	Ni2—N16—H16B	109.6
N15—C15—H15D	109.9	H16A—N16—H16B	108.1
C16—C15—H15D	109.9	C20—N17—Ni2	106.0 (4)
H15C—C15—H15D	108.3	C20—N17—H17A	110.5
N16—C16—C15	113.3 (6)	Ni2—N17—H17A	110.5
N16—C16—H16C	108.9	C20—N17—H17B	110.5
C15—C16—H16C	108.9	Ni2—N17—H17B	110.5
N16—C16—H16D	108.9	H17A—N17—H17B	108.7
C15—C16—H16D	108.9	C17—N18—Ni2	108.2 (4)
H16C—C16—H16D	107.7	C17—N18—H18A	110.1
C20—C17—N18	109.3 (6)	Ni2—N18—H18A	110.1
C20—C17—H17C	109.8	C17—N18—H18B	110.1
N18—C17—H17C	109.8	Ni2—N18—H18B	110.1
C20—C17—H17D	109.8	H18A—N18—H18B	108.4
N18—C17—H17D	109.8	C18—N19—Ni2	110.6 (4)
H17C—C17—H17D	108.3	C18—N19—H19A	109.5
N19—C18—C19	112.6 (6)	Ni2—N19—H19A	109.5
N19—C18—H18C	109.1	C18—N19—H19B	109.5
C19—C18—H18C	109.1	Ni2—N19—H19B	109.5
N19—C18—H18D	109.1	H19A—N19—H19B	108.1
C19—C18—H18D	109.1	C19—N20—Ni2	108.7 (4)
H18C—C18—H18D	107.8	C19—N20—H20A	109.9
C18—C19—N20	114.4 (6)	Ni2—N20—H20A	109.9
C18—C19—H19C	108.7	C19—N20—H20B	109.9
N20—C19—H19C	108.7	Ni2—N20—H20B	109.9
C18—C19—H19D	108.7	H20A—N20—H20B	108.3
N20—C19—H19D	108.7	N12—Ni1—N13	91.7 (2)
H19C—C19—H19D	107.6	N12—Ni1—N10	94.1 (2)
C17—C20—N17	109.5 (6)	N13—Ni1—N10	170.7 (2)
C17—C20—H20C	109.8	N12—Ni1—N11	82.2 (2)
N17—C20—H20C	109.8	N13—Ni1—N11	93.2 (2)
C17—C20—H20D	109.8	N10—Ni1—N11	94.9 (2)
N17—C20—H20D	109.8	N12—Ni1—N14	94.4 (2)
H20C—C20—H20D	108.2	N13—Ni1—N14	81.8 (2)
C8—Mo1—C6	80.5 (3)	N10—Ni1—N14	90.5 (2)
C8—Mo1—C2	72.2 (3)	N11—Ni1—N14	173.8 (2)
C6—Mo1—C2	80.0 (2)	N12—Ni1—N9	171.5 (2)
C8—Mo1—C7	78.1 (3)	N13—Ni1—N9	93.6 (2)
C6—Mo1—C7	73.7 (2)	N10—Ni1—N9	81.6 (2)
C2—Mo1—C7	143.0 (3)	N11—Ni1—N9	90.8 (2)
C8—Mo1—C3	113.5 (2)	N14—Ni1—N9	92.9 (2)
C6—Mo1—C3	143.0 (2)	N15—Ni2—N20	92.3 (2)
C2—Mo1—C3	72.9 (2)	N15—Ni2—N16	79.8 (3)
C7—Mo1—C3	141.0 (2)	N20—Ni2—N16	92.9 (3)
C8—Mo1—C4	140.7 (3)	N15—Ni2—N18	171.3 (3)
C6—Mo1—C4	72.3 (3)	N20—Ni2—N18	92.1 (2)
C2—Mo1—C4	75.5 (3)	N16—Ni2—N18	92.4 (2)
C7—Mo1—C4	118.8 (3)	N15—Ni2—N19	95.6 (3)
C3—Mo1—C4	76.7 (2)	N20—Ni2—N19	82.4 (3)
C8—Mo1—C5	145.4 (2)	N16—Ni2—N19	173.3 (3)
C6—Mo1—C5	108.9 (3)	N18—Ni2—N19	92.5 (3)
C2—Mo1—C5	141.3 (2)	N15—Ni2—N17	94.4 (2)
C7—Mo1—C5	73.1 (2)	N20—Ni2—N17	172.6 (2)
C3—Mo1—C5	79.6 (2)	N16—Ni2—N17	91.4 (2)
C4—Mo1—C5	72.0 (3)	N18—Ni2—N17	81.7 (2)
C8—Mo1—C1	74.3 (2)	N19—Ni2—N17	93.8 (2)
C6—Mo1—C1	144.0 (2)	H1B—O1—H1C	109.5
C2—Mo1—C1	114.9 (2)	H2D—O2—H2A	109.5
C7—Mo1—C1	76.4 (2)	H3B—O3—H3C	109.5
C3—Mo1—C1	72.0 (2)	H4A—O4—H4C	109.5
C4—Mo1—C1	141.3 (2)	H5A—O5—H5C	109.5
C5—Mo1—C1	80.4 (2)		

### Hydrogen-bond geometry (Å, °)

**Table:**

*D*—H···*A*	*D*—H	H···*A*	*D*···*A*	*D*—H···*A*
N9—H9A···N8^i^	0.92	2.27	3.140 (8)	159
N9—H9B···N3^ii^	0.92	2.41	3.179 (7)	141
N10—H10A···N3^iii^	0.92	2.20	3.111 (8)	170
N10—H10B···N5^iii^	0.92	2.58	3.252 (8)	130
N11—H11A···N3^iii^	0.92	2.37	3.219 (8)	153
N12—H12A···N7^iv^	0.92	2.35	3.141 (8)	144
N12—H12B···N5^iii^	0.92	2.25	3.126 (8)	159
N13—H13A···N7^iv^	0.92	2.53	3.434 (8)	167
N13—H13B···N3^ii^	0.92	2.25	3.056 (8)	146
N14—H14A···N8^i^	0.92	2.24	3.066 (8)	149
N14—H14B···O3^iii^	0.92	2.18	3.099 (15)	176
N15—H15A···O1	0.92	2.51	3.290 (16)	143
N15—H15B···O4^v^	0.92	2.48	3.342 (16)	156
N16—H16A···O4	0.92	2.18	3.023 (16)	152
N17—H17A···O4	0.92	2.17	3.040 (17)	157
N17—H17B···O5^v^	0.92	2.28	3.097 (14)	148
N18—H18A···N6^i^	0.92	2.26	3.177 (10)	178
N18—H18B···N4	0.92	2.40	3.216 (8)	148
N19—H19A···N2^i^	0.92	2.63	3.246 (8)	125
N19—H19B···O4^v^	0.92	2.30	3.187 (15)	163
N20—H20A···O3	0.92	2.25	3.165 (12)	172
N20—H20B···N4	0.92	2.43	3.306 (10)	159

Symmetry codes: (i) −*x*+1, *y*+1/2, −*z*+1; (ii) *x*−1, *y*, *z*−1; (iii) −*x*+1, *y*−1/2, −*z*+1; (iv) *x*, *y*, *z*−1; (v) −*x*, *y*+1/2, −*z*+1.
